# Predicting cytotoxicity of PAMAM dendrimers using molecular descriptors

**DOI:** 10.3762/bjnano.6.192

**Published:** 2015-09-11

**Authors:** David E Jones, Hamidreza Ghandehari, Julio C Facelli

**Affiliations:** 1 Department of Biomedical Informatics, University of Utah, Salt Lake City, UT 84112, USA; 2 Department of Bioengineering, University of Utah, Salt Lake City, UT 84112, USA; 3 Department of Pharmaceutics and Pharmaceutical Chemistry, University of Utah, Salt Lake City, UT 84112, USA,; 4 Utah Center for Nanomedicine, Nano Institute of Utah, University of Utah, Salt Lake City, UT 84112, USA

**Keywords:** data mining, machine learning, molecular descriptors, poly(amido amine) dendrimers (PAMAM)

## Abstract

The use of data mining techniques in the field of nanomedicine has been very limited. In this paper we demonstrate that data mining techniques can be used for the development of predictive models of the cytotoxicity of poly(amido amine) (PAMAM) dendrimers using their chemical and structural properties. We present predictive models developed using 103 PAMAM dendrimer cytotoxicity values that were extracted from twelve cancer nanomedicine journal articles. The results indicate that data mining and machine learning can be effectively used to predict the cytotoxicity of PAMAM dendrimers on Caco-2 cells.

## Introduction

In silico approaches, such as data mining and machine learning, have been very successful in medicinal chemistry and are commonly used to guide the design of small pharmaceutical compounds [1]. In contrast, although nanomedicine is a rapidly growing field [2], there have been only a few attempts to use data mining techniques in this field. For instance, Liu et al. analyzed a number of attributes of a variety of nanoparticles in order to predict the 24 hour postfertilization mortality in zebrafish [3]. Horev-Azaria and colleagues used predictive modeling to explore the effect of cobalt–ferrite nanoparticles on the viability of seven different cell lines [4]. Sayes and Ivanov used machine learning to predict the induced cellular membrane damage of immortalized human lung epithelial cells caused by metal oxide nanomaterials [5].

As discussed in a previous paper [6], there are a very limited number of databases compiling the properties of nanomedical relevant compounds. We speculate that this has seriously limited the use of data mining techniques in the field of nanomedicine. However, in the above referenced publication, we demonstrated that natural language processing (NLP) techniques can be effectively used to automatically extract nanoparticle property information from the original literature. Here we argued that this development opens the possibility to explore the use of data mining and chemometric techniques to guide the design of new, more effective treatments using nanoparticles. In this paper we apply the methods of data mining and machine learning to predict the cytotoxicity of poly(amido amine) (PAMAM) dendrimers.

Cytotoxicity was the selected criterion because it is of key concern for the nanoscience and nanomedicine community [7–8], considering that high cytotoxicity is a definitive cause for eliminating a material for potential human applications. Reliable prediction of cytotoxicity using in silico approaches possesses the potential for high payoff in nanomaterial development, allowing the concentration of scarce development resources to be directed towards the synthesis and testing of promising materials with expected low levels of toxicity. Cytotoxicity can be determined by a gamut of in vitro toxicity assays focusing on a number of cellular parameters including cell viability, oxidative stress, genotoxicity, and inflammatory response [9]. In this paper, we focus on the cell viability to characterize cytotoxicity [10].

PAMAM dendrimers are good candidates for a data mining methodological study because they are well documented and have the potential to be highly useful as delivery vectors [11]. These nanoparticles are composed of a central core that is surrounded by concentric shells, thus resulting in their well-defined, highly branched structure [12–13]. The generation of the dendrimer is determined by the number of concentric shells that surround the core of the structure. These polymeric nanoparticles can easily be tailored for specific applications. Benefiting from their characteristic scaffold structures, they have been demonstrated to be suitable carriers for a number of diverse bioactive agents, improving the solubility and bioavailability of poorly soluble ones [14–15]. These particular nanoparticles are also promising for use in the treatment of cancer, including oral formulations. In spite of all the desirable properties of dendrimers, there is a significant setback for their use in biomedicine due to their potential toxicological effects, which depend on the structure that is used. It has been shown that cationic PAMAM dendrimers can have surface charge-, generation-, and concentration-dependent toxicity [16–19].

The goal of this research is to demonstrate that data mining methods like the ones used here can be a presynthesis step to identify nondesirable PAMAM dendrimers that have a substantial probability of high toxicity. It would thus be possible to eliminate them from the early stages of the synthetic development pipeline with reasonable confidence. This technique is not meant to replace cytotoxicity assays in the laboratory, but rather to augment these methods. This method will bolster existing cytotoxicity assays by providing the ability to determine relevant compounds with low cytotoxicity and to eliminate weak-candidate PAMAM dendrimers from synthesis and confirmatory testing. This work also illustrates a proof of concept that data mining and machine learning can be applied to PAMAM dendrimers to predict their biochemical properties. This result could potentially be expanded to other nanomaterials in the future.

## Results and Discussion

Five different analyses were performed to classify a dendrimer as toxic or nontoxic using different combinations of molecular descriptors and experimental conditions. The first analysis utilized all the molecular descriptors available in MarvinSketch (see Experimental section and Table S1 in Supporting Information File 1). The second analysis involved an automatic feature selection method in which the molecular descriptors that were used had a nonzero rank according to the ChiSquaredAttributeEval method in Weka (see details in the Experimental section). The ChiSquaredAttributeEval method determines the rank of an attribute by calculating the chi-squared statistic with respect to the class [20]. The third analysis used only the molecular descriptors selected by expert advice (see details in the Experimental section): molecular weight, atom count, pI, and molecular polarizability. The fourth analysis included the same molecular descriptors used in the second analysis in addition to the experimental concentration (i.e., the amount in mM of PAMAM dendrimer added to the human colon carcinoma Caco-2 cells culture during the cytotoxicity analysis). The final analysis independently assessed the performance of our best method by randomly splitting the dataset into a training set, including 83 of the values, and a test set, including 20 of the values in the dataset.

The results for the first, second, and third analyses performed to classify dendrimers as toxic/nontoxic are presented in Table 1, Table 2, Table 3 and in Supporting Information File 1, Tables S2–S4. The tables list the average precision, recall, F-measure, and mean absolute error for the toxicity class prediction for all classifiers considered here. The tables also contain the accuracy value for the percentage of correctly classified instances. For all analyses, all classifiers consistently had an accuracy at or above 60.2%.

**Table 1:** Results from the 10-fold cross-validation listed by classifier for the first analysis including all molecular descriptors. See Equation 1–4 for the definition of precision, recall, F-measure, and mean absolute error and accuracy.

Classifier	Precision	Recall	F-measure	Mean absolute error	Accuracy

Naive Bayes	0.654	0.660	0.655	0.3370	66.0%
SMO	0.738	0.738	0.725	0.2621	73.8%
J48	0.789	0.748	0.750	0.3077	74.8%
Bagging	0.746	0.738	0.740	0.3211	73.8%
Classification via regression	0.734	0.738	0.730	0.2978	73.8%
Filtered classifier	0.789	0.748	0.750	0.3077	74.8%
LWL	0.775	0.738	0.741	0.2966	73.8%
Decision table	0.678	0.660	0.664	0.3878	66.0%
DTNB	0.691	0.670	0.674	0.3490	67.0%
NBTree	0.696	0.670	0.674	0.3511	67.0%
Random forest	0.736	0.718	0.722	0.3077	71.8%

**Table 2:** Results from the 10-fold cross-validation listed by classifier for the second analysis including the automatically feature-selected molecular descriptors. See Equation 1–4 for the definition of precision, recall, F-measure, and mean absolute error and accuracy.

Classifier	Precision	Recall	F-measure	Mean absolute error	Accuracy

Naive Bayes	0.654	0.660	0.655	0.3370	66.0%
SMO	0.738	0.738	0.725	0.2621	73.8%
J48	0.789	0.748	0.750	0.3077	74.8%
Bagging	0.746	0.738	0.740	0.3211	73.8%
Classification via regression	0.734	0.738	0.730	0.2978	73.8%
Filtered classifier	0.789	0.748	0.750	0.3077	74.8%
LWL	0.775	0.738	0.741	0.2966	73.8%
Decision table	0.678	0.660	0.664	0.3878	66.0%
DTNB	0.691	0.670	0.674	0.3490	67.0%
NBTree	0.696	0.670	0.674	0.3572	67.0%
Random forest	0.736	0.718	0.722	0.2988	71.8%

**Table 3:** Results from the 10-fold cross-validation listed by classifier for the third analysis including the molecular descriptors selected by experts. See Equation 1–4 for the definition of precision, recall, F-measure, and mean absolute error and accuracy.

Classifier	Precision	Recall	F-measure	Mean absolute error	Accuracy

Naive Bayes	0.762	0.748	0.750	0.2822	74.8%
SMO	0.738	0.738	0.725	0.2621	73.8%
J48	0.789	0.748	0.750	0.3077	74.8%
Bagging	0.731	0.718	0.721	0.3217	71.8%
Classification via regression	0.762	0.748	0.750	0.3230	74.8%
Filtered classifier	0.804	0.757	0.760	0.3061	75.7%
LWL	0.834	0.777	0.778	0.3008	77.7%
Decision table	0.658	0.650	0.653	0.3980	65.0%
DTNB	0.658	0.650	0.653	0.3969	65.0%
NBTree	0.722	0.689	0.693	0.3454	68.9%
Random forest	0.758	0.748	0.750	0.2973	74.8%

For the first analysis, Table 1 and Table S2, the J48 and the filtered classifiers show the best results in the 10-fold cross-validation with an accuracy of 74.8%, while bagging, locally weighted learning (LWL), and naive Bayes Tree (NBTree) performed the best with an accuracy of 77.7% in the leave-one-out cross-validation (Table S2). The results from the automatic feature selection analysis, using the ChiSquaredAttributeEval and ranker procedures as the attribute evaluator and search method, respectively, are presented Table 2 and Table S3. These results do not differ drastically from those observed in the first analysis, indicating that the use of automatic feature selection does not improve the classification of toxicity in this study. Alternative automatic feature selection methods using all the WEKA recommended pairings of attribute evaluator and search methods were also tested but did not show any significant improvement in classification prediction performance when using the J48 classifier. These results are presented in Table S7 in Supporting Information File 1. The classification using the features selected by expert advice (Table 3 and Table S4) show that the LWL classifier performed the best with an accuracy of 77.7% in the 10-fold cross-validation. The leave-one-out cross-validation (Table S4) had three classifiers that performed with an accuracy of 78.6% (naive Bayes, bagging, and classification via regression). There is an increase in accuracy across most of the classifiers between the 10-fold and leave-one-out cross-validations. This is an interesting finding because Kohavi noted that k-fold cross-validations typically perform better than leave-one-out cross-validations [21]. This might be an artifact of the dataset not being exactly 50–50 split between toxic and nontoxic samples, thus leading to skewness toward nontoxic predictions.

**Figure 1:**
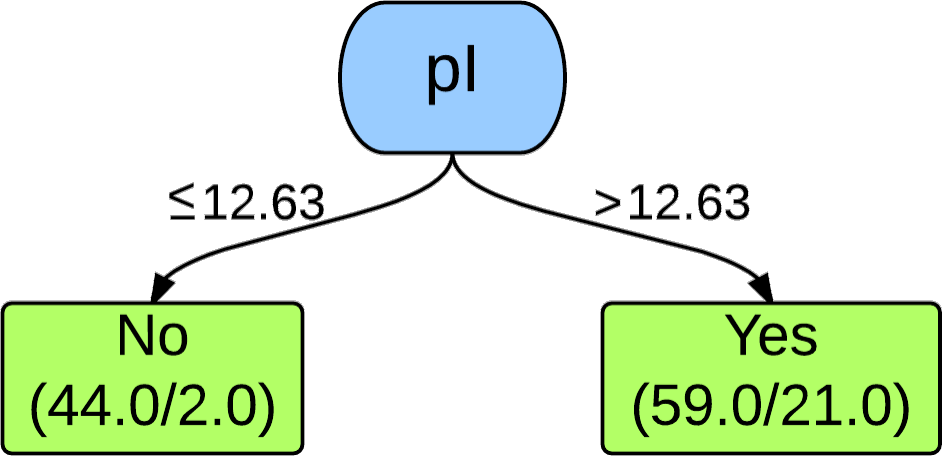
Decision tree for both 10-fold and leave-one-out cross-validation J48 classifier of the first, second, and third analyses. The values indicated on the branches represent the rule or decision used for making the classification. The boxes at the bottom represent the classifications with the number of PAMAM dendrimers classified as such on the left and the number of exceptions (misclassifications) on the right.

The decision tree used by the 10-fold and leave-one-out cross-validation J48 classifiers for the first, second, and third analyses is depicted in Figure 1. As shown in the decision tree, the isoelectric point, pI, is the property that is used to classify the dataset. This property represents the pH at which the net charge of an ionizable molecule is zero. The decision tree indicates that if the pI is greater than 12.63, then the dendrimers are toxic. There are 59 PAMAM dendrimers that are classified as toxic of which 21 are misclassified. If the pI is less than or equal to 12.63, then the dendrimers are classified as nontoxic. There are 44 PAMAM dendrimers classified as nontoxic of which 2 are misclassified.

These results indicate that data mining and machine learning can be implemented to predict the cytotoxicity of PAMAM dendrimers on Caco-2 cells with reasonably high accuracy using only molecular descriptors. The misclassifications observed in Figure 1 are much more significant when examining the dendrimers classified as toxic because almost half of these dendrimers are actually nontoxic. This constitutes a substantial quantity of potentially useful dendrimers that are being ruled out, indicating the necessity for further analysis to decrease the number of false positives.

Table 4 presents the results using the best performing classifiers from the previous section of the analysis using the expert-selected molecular descriptors with the addition of the concentration of dendrimers used in the experiments. No improvement in prediction was observed when using either the filtered or LWL classifiers, but the J48 prediction accuracy of the classification improved to 83.5%. This substantial improvement in the accuracy of the J48 classifications (from 74% to 83.5 %) shows the importance of including the concentration information from the experimental design in addition to the computed molecular descriptors to properly classify compounds as toxic or nontoxic.

**Table 4:** Results from the 10-fold cross-validation listed by classifier for the fourth analysis including the expert-selected molecular descriptors with cytotoxicity concentration. See Equation 1–4 for the definition of precision, recall, F-measure, and mean absolute error and accuracy.

Classifier	Precision	Recall	F-measure	Mean absolute error	Accuracy

Naive Bayes	0.755	0.738	0.741	0.2984	73.8%
SMO	0.738	0.738	0.725	0.2621	73.8%
J48	0.838	0.835	0.836	0.2203	83.5%
Bagging	0.836	0.835	0.835	0.2618	83.5%
Classification via regression	0.742	0.738	0.739	0.3157	73.8%
Filtered classifier	0.804	0.757	0.760	0.3061	75.7%
LWL	0.834	0.777	0.778	0.2995	77.7%
Decision table	0.658	0.650	0.653	0.3980	65.0%
DTNB	0.658	0.650	0.653	0.3969	65.0%
NBTree	0.716	0.689	0.693	0.3347	68.9%
Random forest	0.769	0.767	0.768	0.2483	76.7%

**Figure 2:**
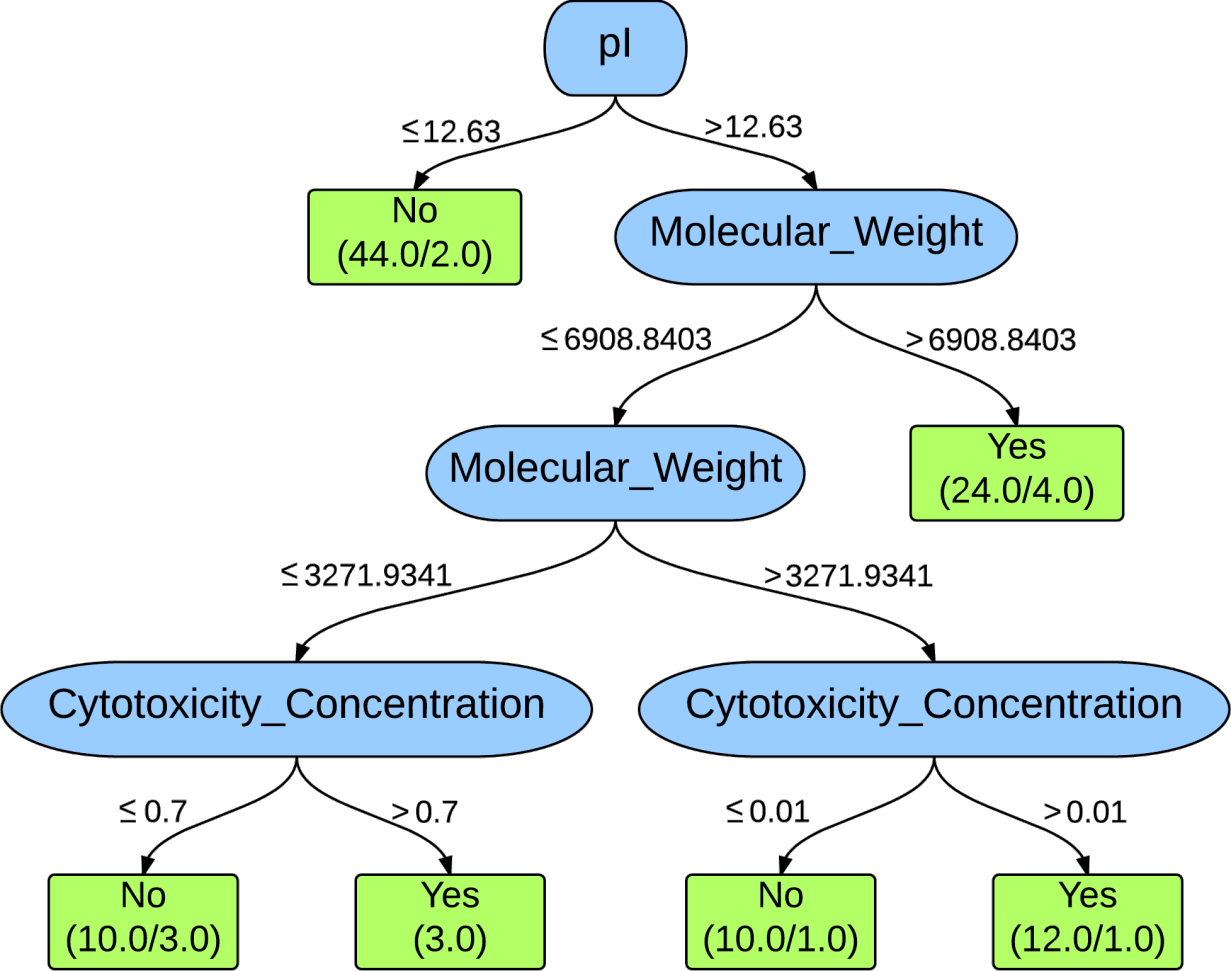
Decision tree for 10-fold cross-validation J48 classifier for the fourth analysis including the molecular descriptors expert-selected with the concentration information of dendrimers used in the experiments. The values present on the branches represent the rule or decision used for making the classification. The boxes at the bottom represent the classifications with the number of PAMAM dendrimers classified as such on the left and the number of exceptions (misclassifications) on the right.

The J48 decision tree for the analysis discussed above is depicted in Figure 2. In this case, the pI, molecular weight, and cytotoxicity concentration are the discriminators in the classification. As can be seen, the feature representing the concentration of dendrimers used in the experiments is present in the decision tree for this analysis. The diagram of the decision trees generated from the J48 classifier illustrates important attributes used in the accurate prediction of toxicity for PAMAM dendrimers. The greatest prediction accuracies were achieved after supplementing the expert-selected features with a descriptor representing the experimental conditions by including the concentration under which the cytotoxicity data was acquired. Figure 2 has the same structure at the top level as Figure 1: when the pI is less than or equal to 12.63, 44 PAMAM dendrimers are classified as nontoxic with an exception of 2 that are misclassified. However, when the pI is greater than 12.63, it leads to other options in the classification of the remaining PAMAM dendrimers. The decision made at the next node is determined for a PAMAM dendrimer molecular weight of ≤6908.8 Da or >6908.8 Da. If the molecular weight is >6908.8 Da, 24 PAMAM dendrimers are classified as toxic with four that are misclassified. If the molecular weight is ≤6908.8 Da, there is another option for the molecular weight being ≤3271.9 Da or >3271.9 Da. The final option can be made considering the concentration target for the desired application of the PAMAM dendrimer. In Figure 2, it can be clearly observed that the number of misclassifications (false positives) has been significantly reduced due to this further analysis (from 21 in Figure 1, to 5 in Figure 2). Due to the significant decrease in false positives, the accuracy of the J48 classifier improved. There was a slight increase in the number of false negatives due to this further analysis (from 2 in Figure 1, to 5 in Figure 2).

The classification scheme in Figure 2 identifies three clusters of viable PAMAM dendrimers that have tolerable levels of cytotoxicity: those with a pI less than or equal to 12.63; those with a pI greater than 12.63, but with molecular weights less than or equal to 3271.9 Da that could be used up to concentrations of less than or equal to 0.7 mM; and those with a pI greater than 12.63, with molecular weights between 6908.8–3271.9341 Da that can be used in formulations requiring concentrations less than or equal to 0.01 mM. When designing novel PAMAM dendrimers, these guidelines could be used for developing viable candidates exhibiting low to no cytotoxicity. This demonstrates the importance of combining experimental conditions with molecular descriptors to achieve the greatest prediction accuracy in the classifiers and to find compounds that may be viable under more restrictive conditions. Another important observation is that the properties present in the decision tree diagrams represent the more general properties of charge, size, and concentration, which have been hypothesized to be the primary causes of cytotoxicity in Caco-2 cells [22].

Table 5 and Table 6 show the data from the external validation study that was performed to further validate the results presented above. For this study, the dataset was randomly split into a training set consisting of 83 cytotoxicity values, and a test set consisting of 20 cytotoxicity values from the original dataset. Table 5 presents the results from the analysis of this test set using all of the molecular descriptors. For all but one of the classifiers, the predicted accuracy was 65.0%, which is slightly lower than the values obtained for the cross-validation analysis, but the LWL classifier performed very well with an accuracy of 95.0%. This is an interesting finding considering that the highest performance of this classifier in the first four analyses was 77.7%. Table 6 shows the data from the analysis of the test set using only the expert-selected features as well as the cytotoxicity concentration data. Again, the LWL classifier performed with an accuracy of 95.0%, thus no improvement was observed in the classification ability of this algorithm between all molecular descriptors and the expert-feature-selected molecular descriptors with cytotoxicity concentration data. There are two algorithms that exhibited a large improvement between Table 5 and Table 6, namely, the naive Bayes and J48 algorithms. Both of these algorithms improved from a prediction accuracy of 65.0% to 90.0%, which is substantially higher than the values obtained in the cross-validation studies.

**Table 5:** Results from the external validation test set analysis listed by classifier using all molecular descriptors. See Equation 1–4 for the definition of precision, recall, F-measure, and mean absolute error and accuracy.

Classifier	Precision	Recall	F-measure	Mean absolute error	Accuracy

Naive Bayes	0.803	0.650	0.617	0.3426	65.0%
SMO	0.803	0.650	0.617	0.3500	65.0%
J48	0.803	0.650	0.617	0.2776	65.0%
Bagging	0.803	0.650	0.617	0.2953	65.0%
Classification via regression	0.803	0.650	0.617	0.3047	65.0%
Filtered classifier	0.803	0.650	0.617	0.2776	65.0%
LWL	0.955	0.950	0.950	0.2510	95.0%
Decision table	0.803	0.650	0.617	0.4206	65.0%
DTNB	0.803	0.650	0.617	0.4182	65.0%
NBTree	0.803	0.650	0.617	0.2945	65.0%
Random forest	0.803	0.650	0.617	0.2784	65.0%

**Table 6:** Results from the external validation test set analysis listed by classifier including the molecular descriptors expert-selected with cytotoxicity concentration. See Equation 1–4 for the definition of precision, recall, F-measure, and mean absolute error and accuracy.

Classifier	Precision	Recall	F-measure	Mean absolute error	Accuracy

Naive Bayes	0.918	0.900	0.900	0.1868	90.0%
SMO	0.803	0.650	0.617	0.3500	65.0%
J48	0.918	0.900	0.900	0.1768	90.0%
Bagging	0.888	0.850	0.849	0.2408	85.0%
Classification via regression	0.803	0.650	0.617	0.3678	65.0%
Filtered classifier	0.803	0.650	0.617	0.2776	65.0%
LWL	0.955	0.950	0.950	0.2467	95.0%
Decision table	0.803	0.650	0.617	0.4206	65.0%
DTNB	0.803	0.650	0.617	0.4182	65.0%
NBTree	0.803	0.650	0.617	0.3082	65.0%
Random forest	0.888	0.850	0.849	0.2187	85.0%

These results indicate that data mining and machine learning can be implemented to accurately predict the cytotoxicity of PAMAM dendrimers on Caco-2 cells. According to Figure 2, the results also indicate that the properties such as charge, size, and the desired concentration of the PAMAM dendrimers in the formulation are the important properties in the prediction of cytotoxicity on Caco-2 cells. We believe that the methods used in this work can be expanded to analyze and predict many other biochemically relevant properties of not only unmodified PAMAM dendrimers but also for surface-modified PAMAM dendrimers. This method will bolster existing cytotoxicity assays by providing the ability to determine relevant compounds with low cytotoxicity for synthesis and confirmatory testing. This thereby reduces the search space necessary for developing biomedically relevant PAMAM dendrimers. This work not only demonstrates a proof of concept that data mining and machine learning can be applied to PAMAM dendrimers to predict the biochemical property of cytotoxicity, but also indicates that further studies including much larger data sets are necessary to develop reliable and robust classification methods that can be apply to a broader set of compounds, cell cultures and experimental designs.

## Conclusion

In this study, classification methods for predicting the Boolean classification of cytotoxicity in Caco-2 cells treated with PAMAM dendrimers were introduced. The results indicate that data mining and machine learning can be used to predict the cytotoxicity of PAMAM dendrimers on Caco-2 cells with good accuracy. In the classification method explored here, it was observed that the properties regarding charge, size, and concentration of the PAMAM dendrimers are the most important properties in the prediction of cytotoxicity and cell viability of Caco-2 cells treated with PAMAM dendrimers. To the authors’ knowledge, these results are the first application of data mining and machine learning to predict the cytotoxicity of PAMAM dendrimers on Caco-2 cells using a classification method.

## Experimental

**Figure 3:**
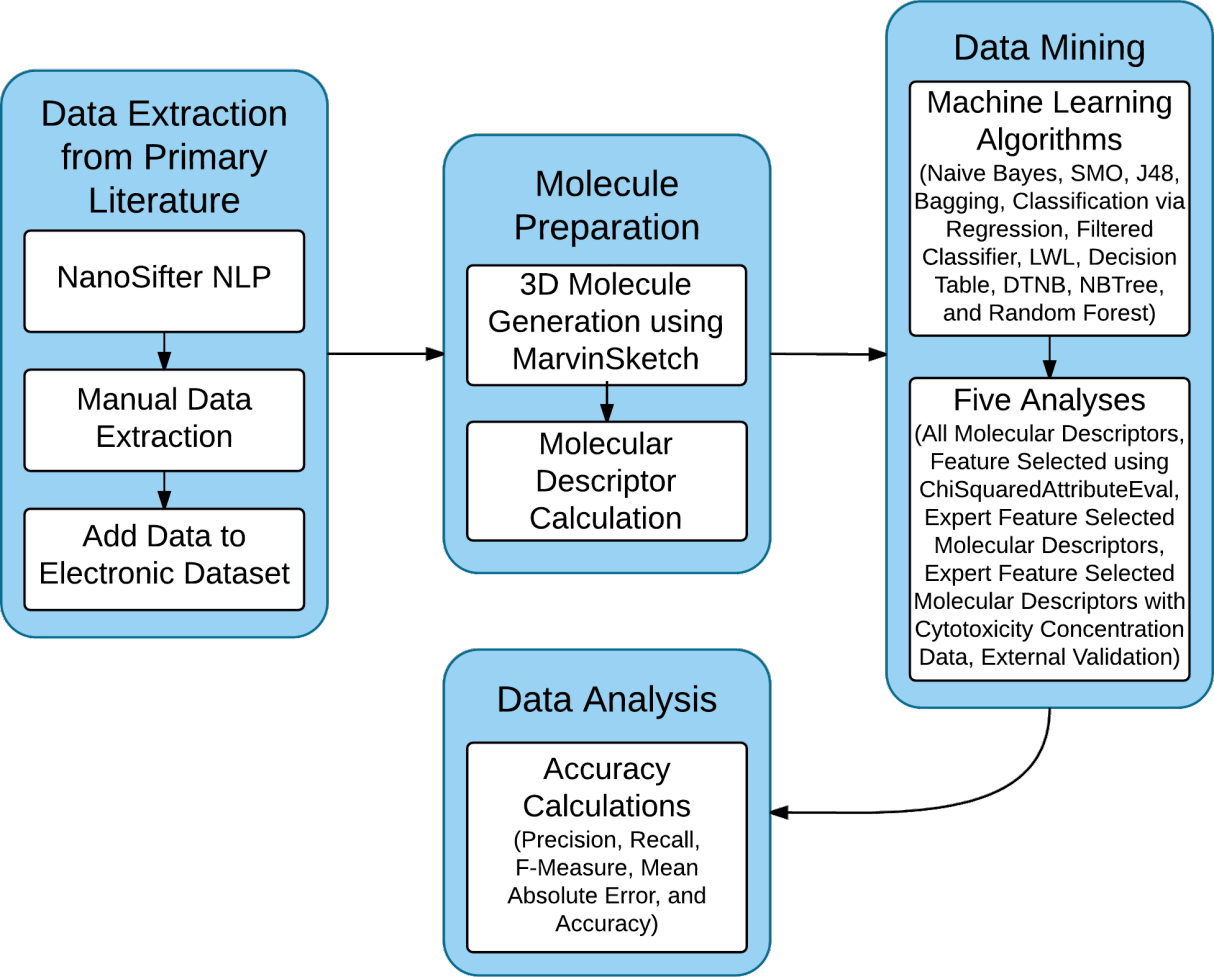
Simplified workflow diagram for the method used in this study.

The overall workflow of the analysis reported in this paper is presented in Figure 3. The details of the different processes are given in the following subsections.

### Nanoparticle selection

The PAMAM dendrimers selected for our study included generations 0, 1, 1.5, 2, 2.5, 3, 3.5, 4, and 4.5 compounds that have been used for transepithelial transport. The full-generation PAMAM dendrimers (generations 0, 1, 2, 3, and 4) are amine- or hydroxy-terminated dendrimers. The half-generation PAMAM dendrimers (generations 1.5, 2.5, 3.5, and 4.5) are carboxyl-terminated dendrimers. For more general property information on the full- and half-generation PAMAM dendrimers, see Table S4 in Supporting Information File 1, which includes the property information for the PAMAM dendrimers analyzed in this study. The toxicity studies used here correspond to assays of these compounds on the human colon carcinoma Caco-2 cell line. The publications containing property data for the nanoparticles selected for this study were gathered from nanomedicine articles available in Scopus and PubMedCentral using the search terms “PAMAM dendrimers AND cytotoxicity AND Caco-2 cells”. In order for the PAMAM dendrimer cytotoxicity values to be considered relevant for extraction, both cell viability and treatment concentration information had to be available in the publication. From this literature corpus, 103 PAMAM dendrimer cytotoxicity values were extracted to be included in this study [23–34]. NanoSifter [6], followed by manual revision, was used to extract the cell viability and cytotoxicity treatment concentration information from the journal articles in the corpus described above.

### Chemical structure rendering and molecular descriptor calculation

The structures of the PAMAM dendrimers were manually constructed using MarvinSketch by ChemAxon [35–36]. There were a total of 10 PAMAM dendrimer structures created for this study. They included generations 0, 1, 1.5, 2, 2.5, 3, 3.5, 4, and 4.5 PAMAM dendrimers. These models include both amine-terminated (full-generation) and carboxyl-terminated (half-generation) structures, as well as one hydroxy-terminated structure (full-generation but hydroxy-terminated). The molecular descriptors for each molecule were calculated using plugins built into MarvinSketch [36]. The list of the 51 molecular descriptors calculated for each molecule is given along with their corresponding definitions in Supporting Information File 1, Table S1. Among these molecular descriptors, there are 42 structural properties (two mass-related, six atom-count-related, seven bond-count-related, four ring-size-related, 13 ring-count-related, and ten other structural properties) and nine chemical properties (five charge-related and four hydrogen-bonding-related properties).

### Data preparation and preprocessing

The data, consisting of the molecular descriptors calculated for all of the molecules considered here and the corresponding cell viability and cytotoxicity data, was uploaded into WEKA [20] to perform the machine learning and data mining analysis using classification methods to discern between toxic and nontoxic compounds. In order to assign a categorical value to each dendrimer cytotoxicity data point, the threshold was established at a cell viability value of 90% (i.e., compounds were considered nontoxic at a certain concentration of PAMAM dendrimer nanoparticles if 90% of the Caco-2 cell population survived after the intervention). Because there is statistical variation in cell viability studies, nontoxic materials can have a few percent above or below 100% cell viability. Hence, the threshold of 90% was set arbitrarily to take into account the usual variability in this type of study.

### Prediction of toxicity using classification methods

Five different analyses were performed to classify a dendrimer as toxic or nontoxic using different combinations of molecular descriptors and experimental conditions. The first analysis utilized all the molecular descriptors. The second analysis involved an automatic feature selection using the ChiSquaredAttributeEval and ranker method built into WEKA, where only molecular descriptors with a nonzero rank were included in this analysis. The molecular descriptors with a nonzero rank were H-bond acceptor sites, pI, logP, Harary index, refractivity, bond count, molecular polarizability, rotatable bond count, atom count, logD, aliphatic bond count, chain bond count, chain atom count, aliphatic atom count, exact mass, molecular weight, Wiener index, Randic index, Szeged index, Wiener polarity, Platt index, H-bond donor count, hyper Wiener index, H-bond donor sites, and H-bond acceptor count. The third analysis used only molecular descriptors selected by expert advice: molecular weight, atom count, pI, and molecular polarizability. In this paper we refer to selected by expert advice as the properties that an experienced researcher in nanocarriers, Dr. Ghandehari, expected to be relevant to predict toxicity based on his own knowledge derived from work is his lab and literature precedents. The fourth analysis included the same molecular descriptors as the ones used in the second analysis and the experimental concentration, i.e., the amount in mM of PAMAM dendrimer added to the Caco-2 cells during cytotoxicity analysis. The fifth analysis was an external validation study in which we randomly selected 20 cytotoxicity values from the original dataset of 103 to create a test set. The remaining 83 cytotoxicity values were used as the training set.

In this work we used the following classifiers: naive Bayes, sequential minimal optimization (SMO), J48, bagging, classification via regression, filtered classifier, LWL, decision table, decision table/naive Bayes (DTNB), NBTree, and random forest. We wanted to explore many modeling methods to provide a wide landscape of available techniques. Since the computational cost is low, there is no strong argument to limit this exploration. Naive Bayes is a Bayesian classifier that uses posterior probability to predict the value of the target attribute [37]. That is, by using a given input attribute, the classifier attempts to find the target attribute value that maximizes the conditional probability of the target attribute. SMO is a support vector machine classifier that globally replaces all values and transforms nominal attributes into binary ones [38]. By default it normalizes all attributes. J48 is a decision tree classifier, which is based on the C4.5 algorithm [39]. This method starts with large sets of cases which belong to known classes, then cases are analyzed for patterns that allow for reliable discrimination of classes. The patterns are represented as models, either in the form of decision trees or sets of if/then rules that can be used to classify new cases. Bagging is a hybrid classification method that creates classes and reduces variance by bagging classifiers [40]. Classification via regression performs its classification by binarizing each class and building one regression model for each class [41]. The filtered classifier is an arbitrary classifier that runs on data passed through an arbitrary filter [20]. LWL uses an instance-based algorithm to assign instance weights [42]. The decision table is a simple decision table majority classifier [43]. DTNB is a decision table/naive Bayes hybrid classifier. During the search, the algorithm determines the need to divide the attributes into two disjoint subsets: one for the decision table, the other for naive Bayes [44]. NBTree is a decision tree/naive Bayes hybrid classifier that builds a decision tree with naive Bayes classifiers at the leaves [45]. All the calculations were performed using WEKA [20].

Two different cross-validation [46] schemes were performed for each classifier. The first one was a 10-fold cross-validation in which the dataset was divided into 10 parts or folds [20]. During each classification run, nine of the folds were used as a training set and one was used as a test set and the results were averaged over the ten runs. The second cross-validation scheme used here was the leave-one-out cross-validation [20]. As this cross-validation method states, one sample is left out as the test set, and the rest of the dataset is the training set. This method runs this through as many iterations as there are samples in the dataset.

The predictions determined by WEKA were evaluated and determined to be true positive, false positive, or false negative by manual inspection. The precision, recall, and F-measure were calculated using the following equations:

[1]
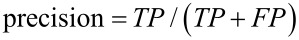


[2]
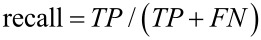


[3]
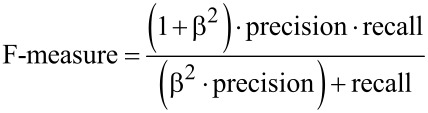


[4]
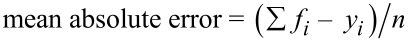


In these equations, TP is true positive, FP is false positive, FN is false negative, and β is the weighting applied to the relationship between precision and recall. The precision and recall were weighted evenly, so β = 1 [6]. The precision, recall, and F-measure of each classifier were calculated for each classification (toxic/nontoxic). Each measure for each classification (toxic/nontoxic) was then averaged. The average value for the precision, recall, and F-measure were recorded. For mean absolute error, *f_i_* is the prediction, *y_i_* is the true value, and *n* is the number of calculated absolute errors.

## Supporting Information

File 1 Supporting Tables.
This document includes all of the tables not present in the text of the document that referenced throughout the document.

File 2 Raw Data.
This is the dataset containing all of the raw data used for all of the analyses in this study.

File 3 SMILES description of all the dendrimers studied here.
