# Supplementary material for: Predicting cytotoxicity of PAMAM dendrimers using molecular descriptors
Source: Beilstein J Nanotechnol. 2015 Sep 11;6:1886–96. doi: 10.3762/bjnano.6.192 (PMC4660915; doi:10.3762/bjnano.6.192)
Supplement: File 1 — Supporting Tables. This document includes all of the tables not present in the text of the document that referenced throughout the document. [file Beilstein_J_Nanotechnol-06-1886-s001.pdf]

Supporting Information

for

Predicting cytotoxicity of PAMAM dendrimers using molecular descriptors

David E. Jones[1], Hamidreza Ghandehari[2,3,4], and Julio C. Facelli[1,4,*]

Address: [1]Department of Biomedical Informatics, University of Utah, Salt Lake City, UT 84112, USA, [2]Department of Bioengineering, University of Utah, Salt Lake City, UT 84112, USA, [3]Department of Pharmaceutics and Pharmaceutical Chemistry, University of Utah, Salt Lake City, UT 84112, USA, and [4]Utah Center for Nanomedicine, Nano Institute of Utah, University of Utah, Salt Lake City, UT 84112, USA

Email: Julio C. Facelli - julio.facelli@utah.edu

[*] Corresponding author

Supporting Tables

Table S1: Listing of the molecular descriptors and their definitions selected from MarvinSketch [4].

Molecular Descriptor	Units	Definition
Molecular Weight	Da	Average molecular mass calculated from the standard atomic weights [1].
Exact Mass	Da	Monoisotopic mass calculated from the weights of the most abundant natural isotopes of the elements [2].
Atom Count		Number of all atoms in the molecule.
pI		Net charge of an ionizable molecule is zero at a certain pH. This pH is called the isoelectric point, also referred to as pI.
logP		The octanol/water partition coefficient, which is used in quantitative structure activity relationships (QSAR) analysis and rational drug design as a measure of molecular hydrophobicity [3].
logD		The octanol-water distribution coefficient, log*D* represents the compounds at any pH value.
Molecular Polarizability	$Å^3$	The electric field generated by partial charges of a molecule spread through intermolecular cavities and the solvent. The induced partial charge (induced dipole) has a tendency to diminish the external electric field. This phenomenon is called polarizability.
Aliphatic Atom Count		Number of atoms in the molecule having no aromatic bond (excluding hydrogens).
Aliphatic Bond Count		Number of non-aromatic bonds in the molecule (excluding bonds of hydrogen atoms).
Aromatic Atom Count		Number of atoms in the molecule having aromatic bonds.
Aromatic Bond Count		Number of aromatic bonds in the molecule.
Asymetric Atom Count		The number of asymmetric atoms (having four different ligands).
Bond Count		Number of bonds in the molecule including bonds of hydrogen atoms.
Chain Atom Count		Number of chain atoms (non-ring atoms excluding hydrogens).
Chain Bond Count		Number of chain bonds (non-ring bonds excluding bonds of hydrogen atoms).
Chiral Center Count		The number of tetrahedral stereogenic centers. This function identifies two chiral centers in 1,4-dimethylcyclohexane,

		which does not contain asymmetric atoms.
Ring Atom Count		Number of ring atoms.
Ring Bond Count		Number of ring bonds.
Rotatable Bond Count		Number of rotatable bonds in the molecule. Unsaturated bonds, and single bonds connected to hydrogens or terminal atoms, single bonds of amides, sulphonamides and those connecting two hindered aromatic rings (having at least three ortho substituents) are considered non-rotatable.
Stereo Double Bond Count		Number of double bonds with defined stereochemistry.
Aliphatic Ring Count		Number of those rings in the molecule that have non-aromatic bonds (SSSR based).
Aromatic Ring Count		Number of aromatic rings in the molecule. This number is calculated from the smallest set of smallest aromatic rings (SSSAR), which might contain rings which are not part of the standard SSSR ring set. As a consequence, the sum of the aliphatic ring count and the aromatic ring count can sometimes be greater than the ring count value. The difference is the signal of a macroaromatic ring system.
Carbo Ring Count		Number of rings containing only carbon atoms.
Carboaliphatic Ring Count		Number of aliphatic rings containing only carbon atoms.
Carboaromatic Ring Count		Number of aromatic rings containing only carbon atoms (SSSAR based).
Fused Aliphatic Ring Count		Number of aliphatic rings having common bonds with other rings.
Fused Aromatic Ring Count		Number of aromatic rings having common bonds with other rings.
Fused Ring Count		Number of fused rings in the molecule (having common bonds).
Hetero Ring Count		Number of rings containing hetero atom(s).
Heteroaliphatic Ring Count		Number of aliphatic heterocycles in the molecule.
Heteroaromatic Ring Count		Number of aromatic heterocycles in the molecule.
Largest Ring Size		Size of the largest ring in the molecule.
Largest Ring System Size		Number of rings in the largest ring system.
Ring Count		Number of rings in the molecule. This calculation is based on SSSR (Smallest Set of Smallest Rings).
Ring System Count		Number of disjunct ring systems.
Smallest Ring Size		Size of the smallest ring in the molecule.

Smallest Ring System Size		Number of rings in the smallest ring system.
Platt Index		Sum of the edge degrees of a molecular graph.
Randic Index		Harmonic sum of the geometric means of the node degrees for each edge.
Harary Index		Half-sum of the off-diagonal elements of the reciprocal molecular distance matrix of the molecule.
Hyper Wiener Index		A variant of the Wiener index.
Szeged Index		The Szeged index extends the Wiener index for cyclic graphs by counting the number of atoms on both sides of each bond (those atoms only which are nearer to the given side of the bond than to the other), and sum these counts.
Wiener Index		The average topological atom distance (half of the sum of all atom distances) in the molecule.
Wiener Polarity		The number of 3 bond length distances in the molecule.
Cyclomatic Number		The smallest number of bonds which must be removed so that no circuit remains. Also known as circuit rank.
Fragment Count		Number of fragments in the sketch.
H-Bond Donor Count		Hydrogen Bond Donor calculates atomic hydrogen bond donor inclination.
H-Bond Donor Sites		Hydrogen Bond Donor calculates atomic hydrogen bond donor inclination.
H-Bond Acceptor Count		Hydrogen Bond Acceptor calculates atomic hydrogen bond acceptor inclination.
H-Bond Acceptor Sites		Hydrogen Bond Acceptor calculates atomic hydrogen bond acceptor inclination.
Refractivity	$10^6[m^3mol^{-1}]$	Molar refractivity is strongly related to the volume of the molecules and to London dispersive forces that has important effect in drug-receptor interaction.

Table S2: Results from the leave-one-out cross-validation listed by classifier of the first analysis including all molecular descriptors.

Classifier	Precision	Recall	F-Measure	Mean Absolute Error	Accuracy
Naïve Bayes	0.738	0.738	0.725	0.2972	73.8%
SMO	0.780	0.767	0.751	0.2330	76.7%
J48	0.748	0.718	0.722	0.3180	71.8%
Bagging	0.800	0.777	0.780	0.3241	77.7%
Classification via Regression	0.780	0.767	0.751	0.2956	76.7%
Filtered Classifier	0.748	0.718	0.722	0.3180	71.8%
LWL	0.834	0.777	0.778	0.2971	77.7%
Decision Table	0.698	0.680	0.683	0.3746	68.0%
DTNB	0.755	0.728	0.732	0.3145	72.8%
NBTree	0.834	0.777	0.778	0.2520	77.7%
Random Forest	0.750	0.738	0.741	0.2874	73.8%

Table S3: Results from the leave-one-out cross-validation listed by classifier of the second analysis including the automatically feature selected molecular descriptors.

Classifier	Precision	Recall	F-Measure	Mean Absolute Error	Accuracy
Naïve Bayes	0.738	0.738	0.725	0.2972	73.8%
SMO	0.780	0.767	0.751	0.2330	76.7%
J48	0.748	0.718	0.722	0.3180	71.8%
Bagging	0.755	0.728	0.732	0.3241	72.8%
Classification via Regression	0.780	0.767	0.751	0.2956	76.7%
Filtered Classifier	0.748	0.718	0.722	0.3180	71.8%
LWL	0.834	0.777	0.778	0.2971	77.7%
Decision Table	0.650	0.641	0.644	0.3736	64.1%
DTNB	0.755	0.728	0.732	0.3145	72.8%
NBTree	0.834	0.777	0.778	0.2520	77.7%
Random Forest	0.750	0.738	0.741	0.2879	73.8%

Table S4: Results from the leave-one-out cross-validation listed by classifier for the third analysis including the molecular descriptors selected by experts.

Classifier	Precision	Recall	F-Measure	Mean Absolute Error	Accuracy
Naïve Bayes	0.796	0.786	0.789	0.2550	78.6%
SMO	0.738	0.738	0.725	0.2621	73.8%
J48	0.748	0.718	0.722	0.3180	71.8%
Bagging	0.813	0.786	0.789	0.3208	78.6%
Classification via Regression	0.796	0.786	0.789	0.2954	78.6%
Filtered Classifier	0.834	0.777	0.778	0.3051	77.7%
LWL	0.834	0.777	0.778	0.3030	77.7%
Decision Table	0.604	0.602	0.603	0.3867	60.2%
DTNB	0.604	0.602	0.603	0.3829	60.2%
NBTree	0.834	0.777	0.778	0.3271	77.7%
Random Forest	0.750	0.738	0.741	0.2865	73.8%

Table S5: Schema describing different properties of the various generations of PAMAM dendrimers [5-12].

Generation	Generation Classification	Surface Group	No. Surface Group	Molecular Weight (Da)	Diameter (nm)
G0	Full	$-NH_2$	4	517	1.5
G1	Full	$-NH_2$	8	1430	2.2
G1.5	Half	-COOH	16	2935	2.2
G2 ($-NH_2$)	Full	$-NH_2$	16	3256	2.9
G2 (-OH)	Full	-OH	16	3272	2.9
G2.5	Half	-COOH	32	6267	3.56
G3	Full	$-NH_2$	32	6909	3.6
G3.5	Half	-COOH	64	12931	3.8
G4	Full	$-NH_2$	64	14215	4.5
G4.5	Half	-COOH	128	26258	4.7

Table S6: Table listing all of the acronyms/abbreviations and their unabbreviated forms.

Acronym/Abbreviation	Unabbreviated Form
NLP	Natrual language processing
PAMAM	Poly(amido amine)
ChiSquaredAttributeEval	Chi squared attribute evaluation
pI	Isoelectric point
LWL	Locally weighted learning
NBTree	Naïve Bayes tree
SMO	Sequential minimal optimization
DTNB	Decision table/Naïve Bayes

Table S7: J48 classification accuracy and RMS when using the features selected using all possible WEKA recommended pairs of Attribute Evaluator and Search Method. The selected features are given in the third column.

Attribute Evaluator	Search Method	Selected Attributes	J48 Accuracy	J48 RMS Error
CfsSubsetEval	BestFirst	pI, logP	74.8	0.4163
CfsSubsetEval	ExhaustiveSearch	DID NOT RUN DUE TO THE LARGE SIZE OF FEATUE SET		
CfsSubsetEval	GreedyStepwise	pI, logP	74.8	0.4163
ChiSquaredAtributeEval	Ranker	H-Bond_Acceptor_Sites, pI, logP, Harary_Index, Refractivity, Bond_Count, Molecular_Polarizability, Rotatable_Bond_Count, Atom_Count, logD, Aliphatic_Bond_Count, Chain_Bond_Count, Chain_Atom_Count, Aliphatic_Atom_Count, Exact_Mass, Molecular_Weight, Wiener_Index, Randic_Index, Szeged_Index, Wiener_Polarity, Platt_Index, H-Bond_Donor_Count, Hyper_Wiener_Index, H-Bond_Donor_Sites, H-Bond_Acceptor_Count	74.8	0.401
ClassifierSubsetEval	GreedyStepwise	NO ATTRIBUTES SELECTED		
ConsistencySubsetEval	GreedyStepwise	pI, logD	74.8	0.4163

CostSensitiveAttributeEval	Ranker	NO ATTRIBUTES SELECTED		
CostSensitiveSubsetEval	GreedyStepwise	NO ATTRIBUTES SELECTED		
FilteredAttributeEval	Ranker	H-Bond_Acceptor_Sites, Harary_Index, logP, pI, Bond_Count, Refractivity, Molecular_Polarizability, Rotatable_Bond_Count, Atom_Count, logD, H-Bond_Donor_Sites, Aliphatic_Atom_Count, Chain_Bond_Count, Aliphatic_Bond_Count, Exact_Mass, Chain_Atom_Count, Molecular_Weight, Szeged_Index, Wiener_Polarity, Randic_Index, Wiener_Index, Platt_Index, H-Bond_Donor_Count, Hyper_Wiener_Index, H-Bond_Acceptor_Count	74.8	0.401
FilteredSubsetEval	GreedyStepwise	pI, logP	74.8	0.4163
GainRatioAttributeEval	Ranker	pI, Hyper_Wiener_Index, logP, Platt_Index, Aliphatic_Atom_Count, Szeged_Index, Aliphatic_Bond_Count, Chain_Atom_Count, Randic_Index, Molecular_Weight,	74.8	0.401

		Chain_Bond_Count, Wiener_Polarity, H-Bond_Donor_Count, Exact_Mass, Wiener_Index, logD, Harary_Index, Bond_Count, Rotatable_Bond_Count, Atom_Count, Molecular_Polarizability, Refractivity, H-Bond_Acceptor_Sites, H-Bond_Donor_Sites, H-Bond_Acceptor_Count		
InfoGainAttributeEval	Ranker	H-Bond_Acceptor_Sites, Harary_Index, logP, pI, Bond_Count, Refractivity, Molecular_Polarizability, Rotatable_Bond_Count, Atom_Count, logD, H-Bond_Donor_Sites, Aliphatic_Atom_Count, Chain_Bond_Count, Aliphatic_Bond_Count, Exact_Mass, Chain_Atom_Count, Molecular_Weight, Szeged_Index, Wiener_Polarity, Randic_Index, Wiener_Index, Platt_Index, H-Bond_Donor_Count, Hyper_Wiener_Index, H-Bond_Acceptor_Count	74.8	0.401

LatentSemanticAnalysis	Ranker	Molecular_Weight, Exact_Mass, Atom_Count, pI, logP, logD, Molecular_Polarizability, Aliphatic_Atom_Count, Aliphatic_Bond_Count, Bond_Count, Chain_Atom_Count, Chain_Bond_Count, Rotatable_Bond_Count, Platt_Index, Randic_Index, Harary_Index, Hyper_Wiener_Index, Szeged_Index, Wiener_Index, Wiener_Polarity, H-Bond_Donor_Count, H-Bond_Donor_Sites, H-Bond_Acceptor_Count, H-Bond_Acceptor_Sites, Refractivity	74.8	0.401
OneRAttributeEval	Ranker	Hyper_Wiener_Index, H-Bond_Donor_Sites, Randic_Index, Harary_Index, Wiener_Index, Platt_Index, Aliphatic_Bond_Count, Szeged_Index, Wiener_Polarity, H-Bond_Donor_Count, Chain_Bond_Count, Aliphatic_Atom_Count, Chain_Atom_Count, H-Bond_Acceptor_Sites, pI, logD, logP, Bond_Count,	74.8	0.401

		Refractivity, Molecular_Polarizability, Atom_Count, Exact_Mass, Rotatable_Bond_Count, Molecular_Weight, H-Bond_Acceptor_Count, Aromatic_Atom_Count, Fused_Aromatic_Ring_Count, Largest_Ring_System_Size, Aromatic_Bond_Count, Ring_System_Count, Ring_Count, Smallest_Ring_Size, Largest_Ring_Size, Fragment_Count, Cyclomatic_Number, Smallest_Ring_System_Size, Asymetric_Atom_Count, Heteroaromatic_Ring_Count, Fused_Ring_Count, Aliphatic_Ring_Count, Carbo_Ring_Count, Carboaliphatic_Ring_Count, Carboaromatic_Ring_Count, Aromatic_Ring_Count, Stereo_Double_Bond_Count, Heteroaliphatic_Ring_Count, Hetero_Ring_Count, Chiral_Center_Count, Ring_Atom_Count, Ring_Bond_Count, Fused_Aliphatic_Ring_Count		
PrincipalComponents	Ranker	Refractivity,	74.8	0.401

		Molecular_Polarizability, Bond_Count, Atom_Count, Wiener_Polarity, Randic_Index, Aliphatic_Bond_Count, Aliphatic_Atom_Count, Chain_Atom_Count, Chain_Bond_Count, Molecular_Weight, Exact_Mass, Platt_Index, H-Bond_Donor_Count, Rotatable_Bond_Count, Harary_Index, H-Bond_Acceptor_Count, H-Bond_Donor_Sites, H-Bond_Acceptor_Sites, logD, Szeged_Index, Wiener_Index, logP, Hyper_Wiener_Index, pI		
ReliefFAttributeEval	Ranker	pI, Hyper_Wiener_Index, logP, H-Bond_Donor_Sites, logD, H-Bond_Donor_Count, H-Bond_Acceptor_Sites, Rotatable_Bond_Count, Bond_Count, Atom_Count, Molecular_Polarizability, Refractivity, Wiener_Polarity, Randic_Index, Aliphatic_Bond_Count, Chain_Bond_Count, Chain_Atom_Count, Aliphatic_Atom_Count, Molecular_Weight,	74.8	0.401

		Exact_Mass, Platt_Index, Harary_Index, H-Bond_Acceptor_Count, Szeged_Index, Wiener_Index		
SVMAttributeEval	Ranker	Hyper_Wiener_Index, H-Bond_Donor_Sites, Randic_Index, Harary_Index, Wiener_Index, Platt_Index, Aliphatic_Bond_Count, Szeged_Index, Wiener_Polarity, H-Bond_Donor_Count, Chain_Bond_Count, Aliphatic_Atom_Count, Chain_Atom_Count, H-Bond_Acceptor_Sites, pI, logD, logP, Bond_Count, Refractivity, Molecular_Polarizability, Atom_Count, Exact_Mass, Rotatable_Bond_Count, Molecular_Weight, H-Bond_Acceptor_Count, Aromatic_Atom_Count, Fused_Aromatic_Ring_Count, Largest_Ring_System_Size, Aromatic_Bond_Count, Ring_System_Count, Ring_Count, Smallest_Ring_Size, Largest_Ring_Size, Fragment_Count, Cyclomatic_Number,	74.8	0.401

		Smallest_Ring_System_Size, Asymetric_Atom_Count, Heteroaromatic_Ring_Count, Fused_Ring_Count, Aliphatic_Ring_Count, Carbo_Ring_Count, Carboaliphatic_Ring_Count, Carboaromatic_Ring_Count, Aromatic_Ring_Count, Stereo_Double_Bond_Count, Heteroaliphatic_Ring_Count, Hetero_Ring_Count, Chiral_Center_Count, Ring_Atom_Count, Ring_Bond_Count, Fused_Aliphatic_Ring_Count		
SymmetricalUncertAttibuteEval	Ranker	pI, logP, Harary_Index, Molecular_Polarizability, Bond_Count, Refractivity, Rotatable_Bond_Count, Atom_Count, H-Bond_Acceptor_Sites, Hyper_Wiener_Index, logD, Aliphatic_Bond_Count, Chain_Bond_Count, Chain_Atom_Count, Exact_Mass, Aliphatic_Atom_Count, Molecular_Weight, Wiener_Index, Randic_Index, Platt_Index, Szeged_Index, H-Bond_Donor_Count, Wiener_Polarity, H-	74.8	0.401

		Bond_Donor_Sites, H-Bond_Acceptor_Count		
WrapperSubsetEval	GreedyStepwise	NO ATTRIBUTES SELECTED		

References

1. Wieser, M. E. *Atomic weights of the elements 2005 (IUPAC Technical Report)"*; Pure Appl. Chem., 2006; pp 2051-2066.

2. Audi, G., *Nuclear Physics* **1995,** *4*, 409-480.

3. Viswanadhan, V. N.; Ghose, A. K.; Revankar, G. R.; Robins, R. K., *Journal of chemical information and computer sciences* **1989,** *29*, 163-172.

4. ChemAxon *Marvin 5.12.4*, 2013.

5. Tomalia, D. A.; Naylor, A. M.; Goddard, W. A., *Angewandte Chemie International Edition in English* **1990,** *29* (2), 138-175.

6. Prieto, M. J.; del Rio Zabala, N. E.; Marotta, C. H.; Carreno Gutierrez, H.; Arevalo Arevalo, R.; Chiaramoni, N. S.; del Valle Alonso, S., *PloS one* **2014,** *9* (2), e90393.

7. Vázquez-Olmos, A.; Díaz, D.; Rodríguez-Gattorno, G.; Saniger-Blesa, J. M., *Colloid and polymer science* **2003,** *282* (9), 957-964.

8. Satoh, K.; Yoshimura, T.; Esumi, K., *Journal of colloid and interface science* **2002,** *255*, 312-322.

9. Kitchens, K. M.; Kolhatkar, R. B.; Swaan, P. W.; Eddington, N. D.; Ghandehari, H., *Pharmaceutical research* **2006,** *23* (12), 2818-2826.

10. Devarakonda, B.; Hill, R. A.; de Villiers, M. M., *International journal of pharmaceutics* **2004,** *284* (1-2), 133-140.

11. Sweet, D. M.; Kolhatkar, R. B.; Ray, A.; Swaan, P.; Ghandehari, H., *Journal of controlled release : official journal of the Controlled Release Society* **2009,** *138* (1), 78-85.

12. El-Sayed, M.; Ginski, M.; Rhodes, C. A.; Ghandehari, H., *journal of Bioactive and Compatible Polymers* **2003,** *18* (1), 7-22.
